# Large Range Curvature Measurement Using FBGs in Two-Core Fiber with Protective Coating

**DOI:** 10.3390/mi15111310

**Published:** 2024-10-28

**Authors:** Ruibin Chen, Lutian Li, Qianqing Yu, Zhijun Luo, Zhenggang Lian, Chuanxin Teng, Hang Qu, Xuehao Hu

**Affiliations:** 1 Research Center for Advanced Optics and Photoelectronics, Department of Physics, College of Science, Shantou University, Shantou 515063, China; 23rbchen@stu.edu.cn (R.C.); 22ltli@stu.edu.cn (L.L.); 2 Yangtze Optical Electronics Co., Ltd., East Lake Hi-Tech Developzone, Wuhan 430205, China; yuqianqing@yoec.com.cn (Q.Y.); luozhijun@yoec.com.cn (Z.L.); lianzhenggang@yoec.com.cn (Z.L.); 3 Guangxi Key Laboratory of Optoelectronic Information Processing, Guilin University of Electronic Technology, Guilin 541004, China; cxteng@guet.edu.cn; 4 Department of Electromagnetism and Telecommunication, University of Mons, Boulevard Dolez 31, 7000 Mons, Belgium

**Keywords:** two-core fiber, FBG, curvature measurements

## Abstract

In this work, we propose a fiber Bragg grating (FBG)-based sensor for curvature measurements. Two gratings are inscribed through the protective coating in a specialty optical fiber using focused femtosecond laser pulses and point-by-point direct writing technology. One grating is inscribed on the central core adjacent to an air channel, while the other is inscribed on the eccentric core. The bending characteristics of the two-core fiber strongly depend on the bending direction due to the asymmetry of the fiber cores. A bending sensitivity of 58 $pm/m^{-1}$ is achieved by the FBG in the eccentric fiber core over the curvature range of 0–50 $m^{-1}$. Temperature and humidity cross-sensitivity could be significantly reduced by analyzing the differences in peak shifts between the two gratings. The sensor features a large sensing range and good robustness due to the presence of its protective buffer coating, which makes it a good candidate for curvature sensing in engineering fields.

## 1. Introduction

The measurement of bending is of great significance in many fields, such as aerospace, robot and structural health monitoring [1,2,3]. Fiber-optic bending sensors, which usually operate by breaking the cylindrical symmetry of modal guidance in fibers, have been extensively investigated based on different configurations, such as lateral-offset Mach–Zehnder interferometers [4,5,6], off-axis fiber Bragg gratings (FBGs) [7,8,9], tilted FBGs [10,11,12] and Long period fiber gratings [13,14,15]. Among them, FBG-based bending sensors are most promising due to the structural simplicity, ease of fabrication, multiplexing, and straightforward demodulation.

FBG-based bending sensors normally require specially designed axially-asymmetrical fibers. In 2015, Hu et al. reported a Bragg grating in D-shaped polymer optical fiber for bend sensing, achieving a bending sensitivity of −28.2 $pm/m^{-1}$ [16]. In 2018, Hou et al. demonstrated a two-dimensional vector-bending sensor using FBGs inscribed in a homogeneous seven-core fiber. The FBGs exhibited high bending sensitivity up to 59.47 $pm/m^{-1}$ [17]. Yong et al. presented a bending sensor with a multimode FBG with asymmetric refractive index distribution in the fiber core [18].

Recently, holey fibers (HFs) have drawn tremendous research attention, as a strong evanescent field can penetrate into to their air holes. They are readily filled with functional fluids, therefore facilitating the detection of ambient changes in temperature, pressure, magnetic field or bending. Jewart et al. [19] presented an FBG-based pressure sensor in a two-hole micro-structured fiber and studied the shift of split birefringent peaks of the FBG in response to external hydrostatic pressure change. In addition, a double-layered Fabry–Perot resonator was formed by a capillary fiber coated with a magnetic gel layer and was used as an all-fiber magnetic field sensor [20]. The good performance and simple fabrication of the HFs allow important applications, such as in-fiber microfluidic and sensing devices [21]. FBG-based HFs applied to bending measurements have been reported with a sensitivity up to 33 $pm/m^{-1}$ by Mao et al. The gratings were inscribed in the two eccentric cores suspended on the hollow fiber [22]. These gratings showed different responsiveness to bending, while showing almost identical responsivity to temperature variations. Thus, temperature cross-sensitivity could be compensated for. However, as both cores were suspended with a distance of ~72 μm on the surface of the hollow fiber, it is somehow difficult to realize the in- and out-coupling of light to the two cores for grating peak interrogation. In addition, the sensing range is limited to 4.76 $m^{-1}$, probably because of the fragile property of the fiber.

In this work, we propose an FBG sensor in a specialty fiber that brings about a very well-conditioned system for the measurement of curvature. The fiber features a central core, an eccentric core and a side hole in its cross-section. The gratings are directly inscribed through the protective coating in the two cores of the fiber using a femtosecond laser and point-by-point (PbP) writing technique. A bending sensitivity up to 58 $pm/m^{–1}$ is obtained by the FBG in the eccentric fiber core over the curvature-radius range of 0–50 $m^{-1}$. A significant advantage of the proposed bending sensor is that its cross-sensitivity to temperature or humidity variations could be compensated for by analyzing the spectral response of the two gratings.

## 2. Curvature Sensor: Basic Structure and Fabrication

In this part, we introduce the structural parameters of the two-core fiber, the inscription process of FBG in the two cores using the femtosecond laser, and the detailed fabrication process of the curvature fiber sensor.

### 2.1. Specific Parameters of Two-Core Fiber

**Figure 1:**
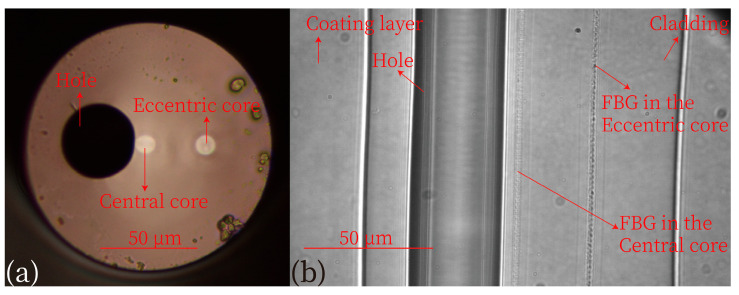
(**a**) Microscopic image of the fiber cross section. (**b**) Microscopic image showing the FBGs PbP-inscribed by the femeosecond laser with a wavelength of 520 nm.

The specialty fiber was fabricated by Yangtze Optical Electronics Co., Ltd., using the regular fiber-preform heat-and-draw route that is also utilized for mass production of standard telecom fibers. Detailed information regarding the fiber fabrication can be found in our previous report [23]. This specialty optical fiber has an outer diameter of ~250 μm, including the protective coating layer, and a bare fiber diameter of 125 μm. It features a central core, an eccentric core, and an air channel with diameters of ~8.1 μm, ~9.2 μm, and ~36 μm, respectively. The distance between the centers of the central core and the eccentric core is 30 μm, and the gap from the edge of the central core to the air channel is ~1.15 μm. A microscopic image of the cross-section of this specialty fiber is shown in Figure 1a.

### 2.2. FBG Design and Fabrication

In general, the two mainstream techniques for FBG fabrication nowadays are UV-laser phase mask lithography and the fs-laser PbP direct writing technique. While the traditional phase mask method is cost effective and highly repeatable, making it ideal for mass production of FBGs, the fs-laser PbP method provides superior thermal stability to the FBG and more flexibility in inscription of customized grating structures (as one can easily adjust the period and index modulation depth of the FBG). Therefore, these two techniques feature exclusive merits depending on different application scenarios. In particular, for writing FBGs in the dual-core fiber, the fs-laser PbP direct writing technique is applicable, since two FBGs with different periods are written into the two individual fiber cores.

The FBG inscription system employs a femtosecond laser (SpOne-8-SHG model from Newport, Irvine, CA, USA), which emits pulses at the wavelength of 520 nm, with a pulse duration of 346 fs, and a maximum repetition rate of 200 kHz. The specialty fiber is immobilized onto a glass substrate with the aid of a rotary fiber clamp (HFR007, Tholabs, Newton, NJ, USA) to ensure both of the cores within the horizontal plane. Particularly, we use a halogen lamp to illuminate one end of the fiber, and use an objective lens (20×) to project the image of the other end onto a screen for verification of the fiber orientation. The orientation of the fiber on the glass substrate can be adjusted by the rotary fiber clamp. Then, the glass substrate is fixed on the multi-axis tilt platform (M-37, Newport), which is mounted on the three-axis precision translation stage (X/Y: XMS100-S, Z: M-VP-5ZA, Newport) of the FBG inscription system. To precisely focus the laser beam onto the two cores of the fiber, a high-resolution oil-immersion objective lens (60×, numerical aperture: 1.42, UPLXAPO60XO, Olympus, Tokyo, Japan) is utilized. During grating fabrication, the reflection spectra of the FBG are monitored in real time by an FBG spectrum interrogator (FS22SI, HBM FiberSensing, Moreira, Portugal) with a wavelength resolution of 1 pm and a scanning rate of 1 Hz. Note that this spectrum interrogator is also equiped with an internal scanning laser source covering the wavelength ranging from 1500 nm to 1600 nm.

The FBGs were inscribed on both the central core and the eccentric core. The laser pulse energy and repetition rate were set to 60 nJ and 50 Hz, respectively. Both FBGs have equal grating lengths of ~5 mm and were positioned in parallel. To obtain different spectral peaks of the gratings and thus avoid interference during measurement, the scanning speeds of the FBGs were set to 26.2 µm/s for the eccentric core and 26.4 µm/s for the central core. This resulted in grating periods of 0.524 µm and 0.528 µm, respectively. A microscopic image of this specialty fiber is shown in Figure 1b, highlighting the locations of the buffer coating layer, the cladding, two cores with gratings, and the air channel.

### 2.3. Design and Fabrication of Curvature Sensor

**Figure 2:**
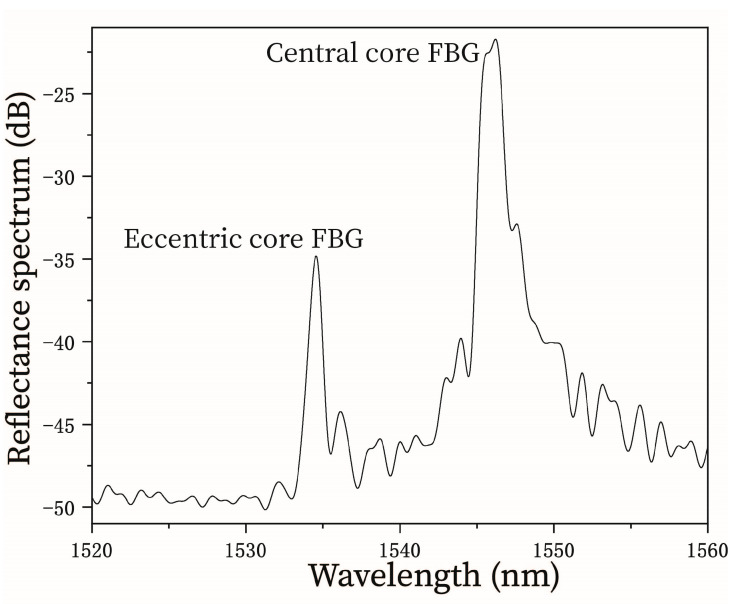
Reflected spectrum of the two FBGs in the eccentric and central core of the specialty fiber.

To fabricate the curvature sensor, an SMF pigtail with a core diameter of 8.2 μm and a cladding diameter of 125 μm was firstly spliced to a multimode fiber (MMF) with a core diameter of 105 μm and a cladding diameter of 125 μm using a fusion splicer (FSM-100P, Fujikura). The MMF was then cleaved to an optimized length of ~1 cm, and acts as a beam expander that enables the guiding light to couple into the two cores of the specialty fiber [23]. The specialty fiber was then fusion spliced to the other end of the MMF. During this discharge process, the electrodes were positioned (using the manual mode of the splicer) predominantly alongside the MMF rather than at the junction interface between the two fibers in order to prevent collapse of the air channel. Figure 2 shows the two reflection peaks at ~1534.6 nm and ~1546.2 nm for the gratings inscribed on the eccentric core and central core, respectively. Note that there is a 15-dB difference in amplitude between these two peaks. This is mainly caused by the different coupling efficiencies between the eccentric core and central core to the lead-in MMF. The coupling efficiencies of the two cores can be balanced by optimizing the fusion splicing of the lead-in MMF to the dual-core fiber, such as by using the off-set splicing technique [24].

## 3. Verification of Sensor Performance

In this section, we first characterize the performance of the curvature sensor featuring a large measuring range and high sensitivity, and then explore the cross impact of temperature and humidity.

### 3.1. Curvature Measurements

**Figure 3:**
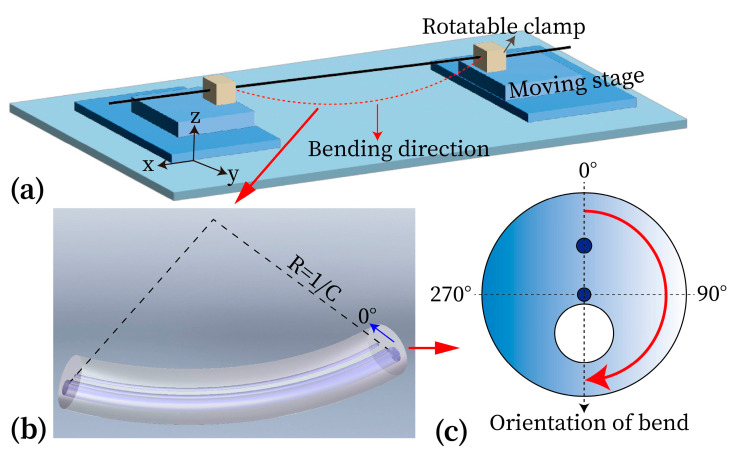
(**a**) Schematic of the experimental set-up for testing bending characteristics; (**b**) illustration of the fiber bending in 0° orientation; (**c**) illustration of four fiber orientations (0°, 90°, 180°, and 270°).

Before conducting the bending measurements, it is necessary to confirm the initial orientation of the specialty fiber by projecting the image of its output end onto a screen using an objective lens. Once the orientation is verified, the responses of the resonant wavelengths of the FBGs to curvature can be investigated with the fiber set in four orthogonal orientations. The curvature responses were measured by suspending the two-core fiber between two moving stages, as shown in Figure 3a. A baffle guides the bent optical fiber into a gap between the two stages, ensuring it remains bent downward in the vertical plane (z direction). The curvature experienced by the fiber can be calculated using the method described in [25]:

(1) $$C=1/R=\sqrt{24x/{\left(L-x\right)}^{3}}$$

where, *R*, *x*, and *L* represent the curvature radius of the bent fiber, the displacement of the moving stage, and the initial length (50 mm) of the sensing fiber. The orientation of the fiber can be adjusted accurately by rotating the fiber clamps, using a 360° rotator with a precision of 1°. To eliminate the influence of temperature and humidity, all bending experiments were conducted at a room temperature of 20 °C and a relative humidity of 50 %RH. The bending response for each FBG was investigated individually in four fiber orientations (0°, 90°, 180°, and 270°), as illustrated in Figure 3b,c. Once the measurements were completed in one orientation, the stage was reset to return the FBG to its initially straight state, and then both fiber clamps were rotated synchronously by 90° for the next round of bending measurements. This process was repeated for all of the four fiber orientations, allowing us to obtain the curvature dependences of the resonant wavelengths for each direction.

**Figure 4:**
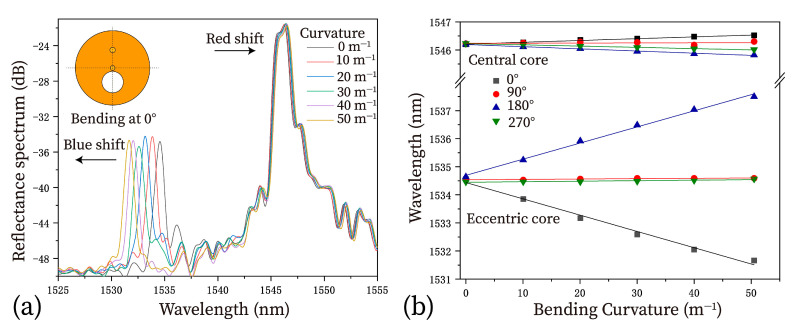
(**a**) The reflected spectrum of the sensor with different curvatures, when the specialty fiber is in the 0° orientation. (**b**) The resonant wavelength dependence on the curvatures for the 0°, 90°,180° and 270° fiber orientations.

For each fiber orientation, the shift in the resonant peak is observed over a bending curvature range of 0 to 50 $m^{–1}$. As shown in Figure 4a, when the two-core fiber is bent at a 0° orientation, the resonant peak of the central core shifts to longer wavelengths, while the resonant peak of the eccentric core exhibits a blue shift. The dependence of resonant wavelengths on curvatures for the four orientations is experimentally demonstrated in Figure 4b. Table 1 presents the linear-fitting results for both FBGs in all four orientations, clearly indicating that all the bending properties exhibit good linear responses, with noticeably different bending sensitivities.

For the FBG in the central core, the sensitivity in all four fiber orientations is similar, at ~5 $pm/m^{–1}$. For the FBG in the eccentric core, its responsivity is different depending on the fiber orientations. This FBG is sensitive to bending for the 0° and 180° fiber orientations, in which the gratings and the fiber’s central axis lie in the bending plane. Additionally, the FBG bending responses for the 0° and 180° fiber orientations are opposite, i.e., a blue shift and a red shift in the resonant peaks corresponding to the bend-induced compression and extension of the FBG, respectively. Experimentally, the FBG in the eccentric core achieved a maximum sensitivity of ~58 $pm/m^{-1}$ for the case of 0° fiber orientation. The measuring range covers from 0 to 50 $m^{-1}$. These results outperformed the sensor performances reported in [22]. The broader curvature measuring range is obtained due to the presence of the fiber coating layer.

For the 90° and 270° fiber orientations, where the plane including the gratings and the fiber’s central axis is perpendicular to the bending plane, the shift of the resonance peak during bending is much smaller when compared to the prior cases. The bending sensitivities were found to be ~1 $pm/m^{-1}$. The insensitive property of the FBG in these orientations is due to the weak compression or extension.

### 3.2. Temperature and Humidity

**Figure 5:**
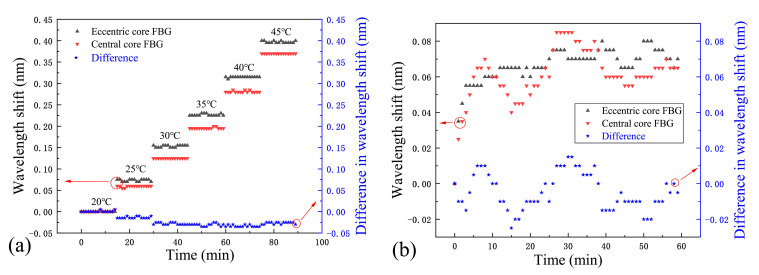
(**a**) Characterization of the thermal stability of the sensor and the difference in spectral shifts of the two FBGs due to temperature variations; (**b**) Characterization of the humidity stability of the sensor and the difference in spectral shift of the two FBGs due to humidity variations.

An important feature of the proposed bending sensor is that its cross-sensitivity to temperature or humidity could be compensated by analyzing the spectral behaviors of the two FBGs. To characterize the cross influence of temperature, the sensor probes were immobilized on the temperature-controlling breadboard (Thorlabs, PTC1/M) with a readout resolution of ±0.001 °C. The influence of the temperature changes was characterized by measuring the resonant wavelength shifts of the two FBGs over a temperature range from 20 °C to 45 °C in 5 °C increments. Each measurement was stabilized for 15 min with the data recorded every minute. The temperature responses of the central core and eccentric core are shown in Figure 5a. The corresponding temperature sensitivities were measured as 14.6 and 16.0 pm/°C, respectively. Moreover, the discrepancies in the spectral shifts of the two FBGs in response to thermal changes within this 25 °C (20–45 °C) window are minute, and the maximum variation is 0.035 nm. Consequently, the temperature-compensated curvature measurements with a higher resolution could be realized by analyzing the differences in the spectral shifts of the two FBGs.

Moreover, the optical fiber’s coating layer is composed of polyacrylate. This coating absorbs or releases moisture in response to changes in the environment’s relative humidity, causing it to expand or contract. The volumetric change induces strain to the fiber due to the close contact between the coating and the fiber [26]. In order to characterize the cross influence of humidity, the probe was immersed in deionized water (considered as humidity over-saturated environment) for 1 h at 20 °C with its spectrum recorded every minute. The experimental results are shown in Figure 5b, from which it can be clearly seen that both of the Bragg peaks redshifted by 0.06 nm within a 5-min immersion, and then tended to be stable afterwards. The discrepancies in the spectral shifts of the two FBGs in response to water absorption are also calculated, and the maximum difference here is only 0.02 nm. Therefore, humidity-compensated curvature measurements can be operated by analyzing the variations in the wavelength shift of the two FBGs.

Generally speaking, the spectral shift of an individual FBG is influenced by mechanical strains caused by a combination of the fiber bending, and temperature and humidity changes. However, the effects of temperature and humidity can be largely compensated for by analyzing the difference in Bragg peak shifts between the two FBGs, resulting in a curvature sensor probe that is insensitive to both temperature and humidity.

### 3.3. Discussion

**Figure 6:**
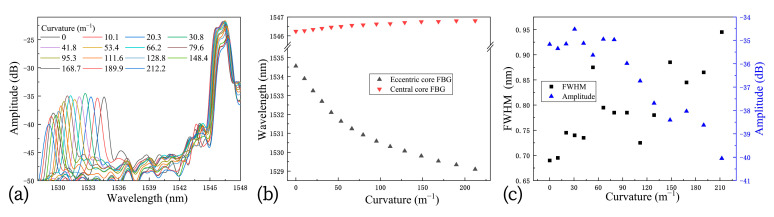
(**a**) Reflection spectra of the fiber sensor as the curvature increased from 0 to ~210 m^−1^, (**b**) spectral shifts of the FBGs in the central core and the eccentric core, (**c**) variations in the FWHM and the amplitude of the reflection peak.

This holey curvature sensor probe is easy to fabricate, and is able to simultaneously couple guiding light into two fiber cores [22]. The coating layer enhances the robustness of the sensor, allowing it to adapt to various complex environments and providing a great measuring range for curvature. The FBG on the eccentric fiber core is particularly sensitive to compression or stretching, thus giving the sensor a high curvature sensitivity. As shown in Table 2, we compared the proposed sensor with several recently reported studies in terms of structure, curvature sensitivity, and measuring range. Although the sensitivity of the proposed sensor is lower than that of the multicore [26] and two-core [27] fiber sensors, it incorporates a fluidic channel, making it suitable for multi-parameter applications beyond just bending measurements. Most notably, the sensing range of this sensor is more than ten times greater than that in previous work, due to the protective polymer coating that reduces the risk of fiber breakage. Note also that the sensing range of the sensor could be further expanded. We carried out experiments to measure the reflection spectra of the sensor at the 0° orientation with curvatures ranging from 0 to ~210 m^−1^. We experimentally found that, when the curvature is greater than 60 m^−1^, using Equation (1) to calculate the curvature would result in relatively large errors. Thus, we had to exert the 3-point-fitting technique to estimate the curvature [27]. Briefly speaking, we took photos of the bent fiber, and three points on the fiber edge are chosen to fit the arc shape of the fiber in order to calculate the curvature radius. The reflection spectra of the sensor probe are shown in Figure 6a, as the curvature increases from 0 to ~210 m^−1^. The corresponding spectral shifts of the FBGs in the central core and in the eccentric core are presented in Figure 6b. While the spectral shifts of FBGs feature clear linearity in response to the curvatures in the range from 0 to 50 m^−1^, pronounced nonlinearity could be observed for the curvature greater than 60 m^−1^. Moreover, due to the bending of the fiber, the full width at half maximum (FWHM) of the spectra tended to increase (Figure 6c), and the reflection amplitude would gradually decrease. This somehow made it more difficult to find the precise locations of the Bragg peak wavelengths. Therefore, in this paper, we only fully characterized the sensor with the measuring range from 0 to 50 m^−1^; however, technically speaking, the sensor dynamic range could be extended to more than 200 m^−1^ without a fiber breakage.

Finally, we would like to mention that the polyacrylate coating may experience degradation (due to factors such as hydrolysis, photo degradation, wind erosion and dissolution) over long-term usage, especially for outdoor applications [28]. While studying the degradation of the coating is not the main purpose of this paper, we note that the proposed sensor probe, when operated for long-term outdoor applications, should be further protected with additional encapsulations.

**Table 2:** Comparison with other FBG bending sensors in terms of sensitivity and curvature range.

Sensor Structures	Holey Fiber	$\mathrm{Curvature}\;\mathrm{Sensitivity}\;(pm/m^{–1}$)	$\mathrm{Curvature}\;\mathrm{Range}\;(m^{-1}$)	Ref.
FBGs in multicore fiber	No	128	0–1.896	[29]
FBGs in two-core Rectangular fiber	No	128	1.2–3	[30]
FBGs in two-core fiber	Yes	33	0–4.759	[22]
FBGs (central core and eccentric core)	Yes	58	0–50	This work

## 4. Conclusions

In summary, we successfully proposed and demonstrated a novel two-core fiber sensor based on FBGs for high-precision curvature measurements. By inscribing two FBGs in a specially designed two-core fiber, we achieved a bending sensitivity of up to 58 $pm/m^{-1}$, covering a bending range from 0 to 50 $m^{-1}$. At the same time, the sensor had compensated cross-sensitivity to environmental temperature and humidity, which opened up the possibility of its application in complex environments. We believe that the proposed sensor would lead to niche engineering applications, where bending/curvature measurements are relevant. Future work will focus on further optimizing the sensor design, improving its long-term stability and reliability, and exploring its performance in practical applications.

## Figures and Tables

**Table 1:** Sensitivity and $R^{2}$ of FBG in the center core and the eccentric core in four fiber orientations.

	Eccentric Core FBG		Central Core FBG	
Orientation	$\mathrm{Sensitivity}\;(pm/m^{-1}$)	$R^{2}$	$\mathrm{Sensitivity}\;(pm/m^{-1}$)	$R^{2}$
$0^{{}^{\circ}}$	−58	98.6	6.42	99.2
$90^{{}^{\circ}}$	1.12	85.3	2.51	96.9
$180^{{}^{\circ}}$	57.47	99.4	−7.71	98.2
$270^{{}^{\circ}}$	1.68	83.2	4.48	98.9

## Data Availability

The original contributions presented in the study are included in the article; further inquiries can be directed to the corresponding authors.
